# 伴1号染色体异常骨髓增生异常肿瘤患者临床特征及预后分析

**DOI:** 10.3760/cma.j.cn121090-20250820-00393

**Published:** 2026-01

**Authors:** 征凌 匡, 冰 李, 铁军 秦, 泽锋 徐, 士强 曲, 承文 李, 丽娟 潘, 清妍 高, 蒙 焦, 玉娇 贾, 琦 孙, 志坚 肖

**Affiliations:** 1 中国医学科学院血液病医院（中国医学科学院血液学研究所），血液与健康全国重点实验室，国家血液系统疾病临床医学研究中心，细胞生态海河实验室，天津 300020 State Key Laboratory of Experimental Hematology, National Clinical Research Center for Blood Diseases, Haihe Laboratory of Cell Ecosystem, Institute of Hematology & Blood Diseases Hospital, Chinese Academy of Medical Sciences & Peking Union Medical College, Tianjin 300020, China; 2 天津医学健康研究院，天津 301600 Tianjin Institutes of Health Science, Tianjin 301600, China

**Keywords:** 骨髓增生异常肿瘤, 1号染色体, 基因突变, 预后, Myelodysplastic neoplasms, Chromosome 1, Mutational profile, Prognosis

## Abstract

**目的:**

分析伴1号染色体异常的骨髓增生异常肿瘤（MDS）患者临床和实验室特征及预后。

**方法:**

收集2016年8月至2024年6月于中国医学科学院血液病医院确诊的1 498例MDS初治患者病例资料，回顾性分析伴1号染色体异常的MDS患者临床特征、分子学等实验室特征以及总生存（OS）情况。

**结果:**

伴1号染色体异常患者共128例（8.54％），其中1q三体最为常见（85例，66.4％）。非1q三体患者较1q三体患者的骨髓原始细胞比例（5.0％对2.5％，*P*＝0.030）、TP53突变检出率（30.2％对12.9％，*P*＝0.033）及TP53中位等位基因突变频率（VAF）（46.7％对19.7％，*P*＝0.034）均显著增高。伴1号染色体异常患者的OS时间［29（95％ *CI*：17～41）个月］与不伴1号染色体异常患者［34（95％ *CI*：25～43）个月］相比差异无统计学意义（*P*＝0.800）。伴1号染色体异常患者中，1q三体与非1q三体两组患者中位OS时间分别为58（95％ *CI*：24～107）个月和10（95％ *CI*：5～15）个月，差异有统计学意义（*P*＝0.005）。多因素分析示，原始细胞增多（IB）（*HR*＝2.23，95％ *CI*：1.10～4.54，*P*＝0.027）、SF3B1突变（*HR*＝5.61，95％ *CI*：2.06～15.29，*P*＝0.001）是影响伴1号染色体异常MDS患者生存的独立不良预后因素。

**结论:**

伴1号染色体异常的MDS患者中，1q三体与低原始细胞和低TP53突变检出率及VAF值相关，1q三体组OS时间显著延长。IB、SF3B1突变是影响伴1号染色体异常MDS患者生存的独立不良预后因素。

## Abstract (en)

**Objective:**

To characterize the clinical and molecular features and evaluate the prognostic outcomes of patients with myelodysplastic neoplasms（MDS）with chromosome 1 abnormalities.

**Methods:**

We retrospectively analyzed 1 498 newly diagnosed MDS patients treated at the Institute of Hematology, Chinese Academy of Medical Sciences from August 2016 to June 2024. The clinical features, molecular characteristics, and overall survival（OS）of patients with chromosome 1 abnormalities were analyzed.

**Results:**

Chromosome 1 abnormalities were identified in 128 patients（8.54％）, with 1q trisomy representing the most frequent cytogenetic alteration（85 cases, 66.4％）. Compared to 1q trisomy patients, non-1q trisomy patients demonstrated significantly higher bone marrow blast percentages（5.0％ *vs* 2.5％, *P*＝0.030）, TP53 mutation rates（30.2％ *vs* 12.9％, *P*＝0.033）, and median TP53 variant allele frequencies（VAF）（46.7％ *vs* 19.7％, *P*＝0.034）. No significant difference in OS was observed between patients with and without chromosome 1 abnormalities［29（95％ *CI*: 17–41）months *vs* 34（95％*CI*: 25–43）, *P*＝0.800］. However, among patients with chromosome 1 abnormalities, the 1q trisomy patients showed markedly superior median OS compared to the non-1q trisomy patients［58（95％ *CI* : 24–107）*vs* 10（95％ *CI*: 5–15）months, *P*＝0.005］. Multivariate analysis identified increased blast（IB）counts（*HR*＝2.23, 95％ *CI*: 1.10–4.54, *P*＝0.027）and SF3B1 mutations（*HR*＝5.61, 95％ *CI*: 2.06–15.29, *P*＝0.001）as independent adverse prognostic factors for survival in this cohort.

**Conclusion:**

Among MDS patients with chromosome 1 abnormalities, 1q trisomy is associated with lower blast counts and reduced TP53 mutation rates/VAF, correlating with significantly improved OS. IB and SF3B1 mutations independently predict poorer survival outcomes in this patient population.

骨髓增生异常肿瘤（MDS）是一组起源于造血干/祖细胞的髓系肿瘤，表现为外周血血细胞减少、骨髓和外周血一系或多系发育异常及高风险向急性髓系白血病转化[1]。恶性血液病中1号染色体克隆性异常多发生于急性白血病（AL）、MDS及多发性骨髓瘤（MM）中，其特征性表现主要为1号染色体与其他染色体之间发生易位和形成1q三体[2]。1q三体是1号染色体长臂重复拷贝所致，在血液髓系肿瘤中以MDS最常见[3]。目前，国内关于MDS中1号染色体异常的报道尚不多见。本研究我们回顾性分析了我院收治的伴1号染色体异常的128例MDS患者的临床、实验室特征，探讨其生存及预后影响因素，现报道如下。

## 病例与方法

1. 病例资料：本研究为回顾性队列研究，2016年8月至2024年6月于中国医学科学院血液病医院MDS和骨髓增殖性肿瘤（MPN）诊疗中心确诊、具有完整病历资料的初治成人MDS患者共1 498例，其中伴1号染色体异常128例（8.54％）纳入研究。所有患者均符合WHO（2022）诊断标准[4]。本研究通过中国医学科学院血液病医院伦理委员会审批（批件号：IIT2021029-EC-1），并豁免知情同意。

2. 染色体核型分析：短期培养法常规制备染色体标本，R显带法分析核型，根据《人类细胞遗传学国际命名体制（ISCN2016）》描述核型，按照修订的国际预后积分系统（IPSS-R）对染色体核型进行预后分组[5]。

3. 二代测序检测基因突变：分离患者骨髓单个核细胞提取基因组DNA，针对包括ASXL1、SF3B1、RUNX1等基因在内的141个或267个血液肿瘤相关基因编码序列进行测序，等位基因突变频率（VAF）≥2％的基因突变纳入分析，利用CCDS、dbSNP（v138）、COSMIC、PolyPhen-2等数据库进行生物信息学分析，筛选致病性基因突变位点。具体方法参见本中心此前发表文献[6]–[7]。

4. 随访：随访截止时间为2024年10月25日。随访资料来源于住院和门诊病历及电话随访记录。128例患者中位随访时间为21（*IQR*：15.4～26.6）个月，共10例（7.8％）患者失访。总生存（OS）时间指自诊断日期到死亡或造血干细胞移植或末次随访日期。

5. 统计学处理：统计分析利用SPSS 26.0完成。非正态分布的计量资料以“中位数（*IQR*）”描述，采用Mann-Whitney *U*检验进行组间比较；率的比较采用卡方检验或Fisher精确概率法。基因间的关联分析通过多重假设检验校正的Fisher确切检验进行评估（*Q*<0.05为差异有统计学意义）。采用Kaplan-Meier法绘制生存曲线，Cox比例风险回归模型进行预后因素分析，预后因素分析中将连续变量转化为二分类变量。双侧*P*<0.05被认为差异具有统计学意义。应用R 4.3.2绘图。

## 结果

一、临床特征

1. 一般特征：128例伴1号染色体异常MDS患者中，男79例（61.7％），女49例（38.3％），中位年龄55.5（48～62）岁。依据WHO 2022诊断标准：MDS伴低原始细胞和孤立5q缺失（MDS-5q）2例（1.6％）、MDS伴低原始细胞（LB）和SF3B1突变（MDS-SF3B1）7例（5.5％）、MDS伴TP53双等位基因失活（MDS-biTP53）22例（17.2％）、MDS伴低原始细胞（MDS-LB）35例（27.3％）、低增生MDS（MDS-h）13例（10.2％）、MDS伴原始细胞增多-1（MDS-IB1）24例（18.8％）、MDS伴原始细胞增多-2（MDS-IB2）16例（12.5％）、MDS伴纤维化（MDS-f）6例（4.7％）。根据IPSS-R预后分组，较高危组（高危/极高危）的患者共80例（62.5％）。根据纳入分子遗传学的IPSS（IPSS-M）预后分组，较高危组（中高危/高危/极高危）的患者共98例（76.6％）。1q三体最为常见（85例，66.4％）。1q三体与非1q三体患者相比，仅HGB（*P*＝0.034）、骨髓原始细胞比例（*P*＝0.030）组间差异有统计学意义（表1）。而性别、ANC、PLT、WHO 2022诊断分型等两组差异均无统计学意义。

**表1:** 128例伴1号染色体异常骨髓增生异常肿瘤患者的临床和实验室特征

特征	全部（128例）	非1q三体（43例）	1q三体（85例）	*P*值
年龄［岁，*M*（*IQR*）］	55.5（48.0，62.0）	57.0（51.0，64.5）	54.0（45.0，61.0）	0.069
男性［例（％）］	79（61.7）	26（60.5）	53（62.4）	0.988
ANC［×10^9^/L，*M*（*IQR*）］	0.94（0.58，1.65）	0.93（0.63，1.79）	0.95（0.57，1.59）	0.427
HGB［g/L，*M*（*IQR*）］	79.5（64，100）	75（61，86）	84（66，105）	0.034
PLT［×10^9^/L，*M*（*IQR*）］	64.5（26，122）	73（30，128）	64.0（26，117）	0.532
网织红细胞百分比［％，*M*（*IQR*）］	1.73（1.06，2.27）	1.83（0.90，2.41）	1.69（1.19，2.19）	0.662
MCV［fl，*M*（*IQR*）］	101（93，107）	97（91，105）	101（95，107）	0.307
LDH［U/L，*M*（*IQR*）］	227（175，292）	232（166，304）	226（176，287）	0.825
血清铁蛋白［µg/L，*M*（*IQR*）］	349（161，724）	407（241，719）	333（147，736）	0.166
EPO［U/L，*M*（*IQR*）］	201（56，758）	207（100，760）	179（48，758）	0.227
骨髓原始细胞比例［％，*M*（*IQR*）］	3.50（1.00，7.50）	5.00（2.75，9.25）	2.50（1.00，6.50）	0.030
骨髓纤维化2～3级［例（％）］	21（16.5）	10（23.3）	11（13.1）	0.228
WHO 2022诊断分型［例（％）］				0.185
MDS-5q	2（1.6）	0（0）	2（2.35）	
MDS-SF3B1	7（5.5）	3（7.0）	4（4.71）	
MDS-biTP53	22（17.2）	12（27.9）	10（11.8）	
MDS-LB	35（27.3）	8（18.6）	27（31.8）	
MDS-h	13（10.2）	4（9.30）	9（10.6）	
MDS-IB1	24（18.8）	6（14.0）	18（21.2）	
MDS-IB2	16（12.5）	5（11.6）	11（12.9）	
MDS-f	6（4.7）	4（9.30）	2（2.35）	
复杂核型［例（％）］	62（48.4）	23（53.5）	39（45.9）	0.531
IPSS-R染色体分组［例（％）］				0.131
极好/好/中等	70（54.7）	19（44.2）	51（60.0）	
差/极差	58（45.3）	24（55.8）	34（40.0）	
IPSS-R预后分组［例（％）］				0.161
极低危/低危/中危	48（37.5）	12（27.9）	36（42.4）	
高危/极高危	80（62.5）	31（72.1）	49（57.6）	
IPSS-M预后分组［例（％）］				0.114
极低危/低危/中低危	30（23.4）	6（14.0）	24（28.2）	
中高危/高危/极高危	98（76.6）	37（86.0）	61（71.8）	
TP53突变［例（％）］	24（18.8）	13（30.2）	11（12.9）	0.033
SF3B1突变［例（％）］	14（10.9）	5（11.6）	9（10.6）	1.000
RUNX1突变［例（％）］	17（13.3）	4（9.30）	13（15.3）	0.504
ASXL1突变［例（％）］	24（18.8）	8（18.6）	16（18.8）	1.000
U2AF1突变［例（％）］	16（12.5）	2（4.6）	14（16.5）	0.104
DNMT3A突变［例（％）］	9（7.0）	6（14.0）	3（3.5）	0.060
NRAS突变［例（％）］	10（7.8）	3（7.0）	7（8.2）	1.000
CBL突变［例（％）］	5（3.9）	1（2.3）	4（4.7）	0.663
EZH2突变［例（％）］	7（5.5）	0（0）	7（8.2）	0.094
ETV6突变［例（％）］	6（4.7）	1（2.3）	5（5.9）	0.663

**注** MCV：平均红细胞体积；LDH：乳酸脱氢酶；EPO：促红细胞生成素；MDS-5q：MDS伴低原始细胞和孤立5q缺失；MDS-SF3B1：MDS伴低原始细胞和SF3B1突变；MDS-biTP53：MDS伴TP53双等位基因失活；MDS-LB：MDS伴低原始细胞；MDS-h：低增生MDS；MDS-IB1：MDS伴原始细胞增多-1；MDS-IB2：MDS伴原始细胞增多-2；MDS-f：MDS伴纤维化；IPSS-R：修订的国际预后积分系统；IPSS-M：纳入分子遗传学的国际预后积分系统

2. 患者细胞遗传学特征：128例伴1号染色体异常患者中，复杂核型检出率为48.4％（62/128）。根据IPSS-R细胞遗传学预后分组，染色体预后较差（差/极差）的患者共58例（45.3％）。1q三体与非1q三体患者相比，复杂核型检出率、IPSS-R细胞遗传学分组差异均无统计学意义。

3. 分子遗传学特征：128例患者均进行了二代测序基因突变检测，基因突变图谱见图1。其中检出率较高的依次为ASXL1（24例，19％）、TP53（24例，19％）、RUNX1（17例，13％）、U2AF1（16例，12％）、SF3B1（14例，11％）、NRAS（14例，11％）、DNMT3A（14例，11％）。与非1q三体患者相比，1q三体患者TP53基因突变检出率显著减低［11例（12.9％）对13例（30.2％），*P*＝0.033］（表1），且1q三体患者TP53 VAF低于非1q三体患者［19.7％（4.2％～41.7％）对46.7％（26.9％～57.0％），*z*＝−2.115，*P*＝0.034］（图2）。

**图1:**
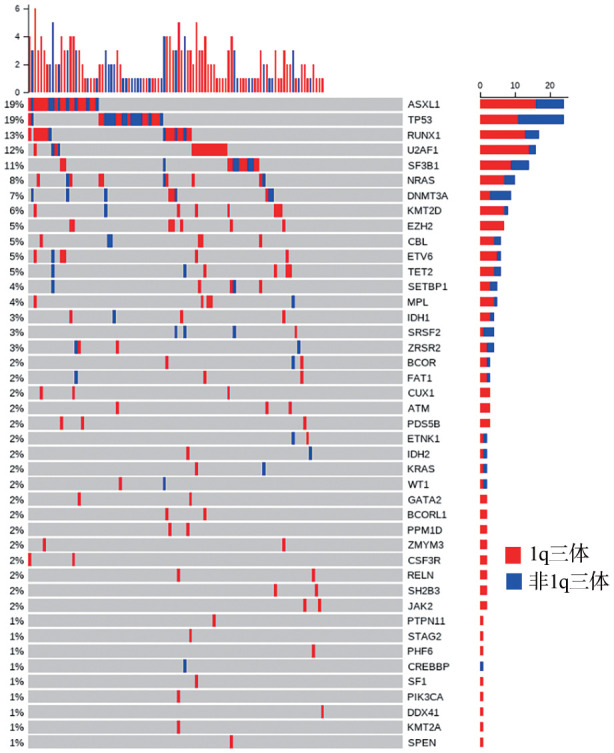
128例伴1号染色体异常骨髓增生异常肿瘤患者基因突变谱系特征

**图2:**
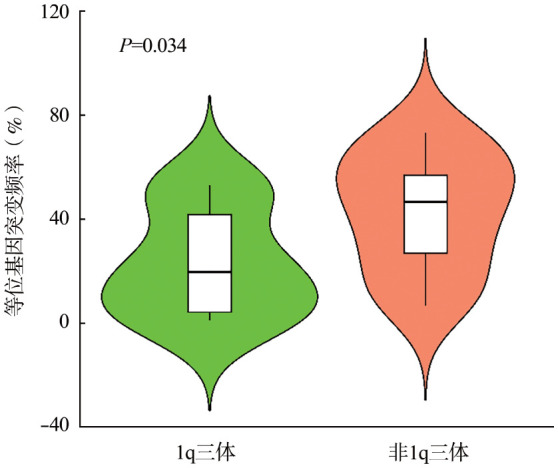
1q三体（11例）与非1q三体（13例）组骨髓增生异常肿瘤患者的TP53等位基因突变频率

4. 常见突变基因与临床特征的关联分析：将突变检出率>5％的基因与常见的临床预后参数进行关联分析。采用Fisher确切检验并进行FDR校正，结果显示DNMT3A和RUNX1突变更常见于老年（≥60岁）患者，而在PLT≥100×10^9^/L患者中较少见。此外，TP53突变在HGB≥100 g/L及1q三体患者中较少见（图3）。

**图3:**
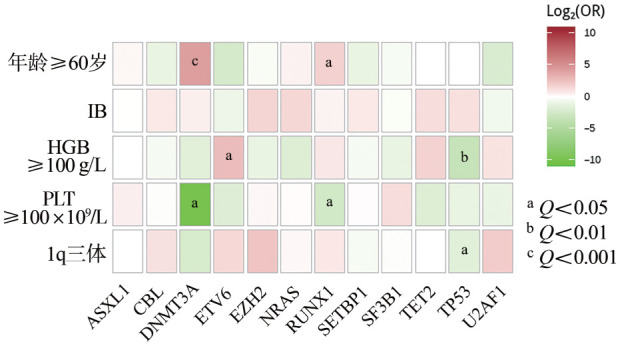
伴1号染色体异常骨髓增生异常肿瘤患者重现性突变基因与临床参数之间的配对关联 **注** IB：骨髓原始细胞≥5％或外周血原始细胞≥2％或出现Auer小体

二、生存分析

对有随访结果的118例1号染色体异常患者进行生存分析，全部患者OS时间为29（95％*CI*：17～41）个月，其中1q三体与非1q三体两组中位OS时间分别为58（95％*CI*：24～107）个月和10（95％*CI*：5～15）个月（*P*＝0.005）。进一步分别按是否为IB、TP53是否突变行亚组分析：在LB亚组中，1q三体患者中位OS时间有长于非1q三体患者［未达到对29（95％*CI*：2～56）个月，*P*＝0.090］的趋势，而在IB亚组中未观察到；在TP53未突变亚组中，1q三体患者有更长的中位OS时间趋势［58（95％*CI*：10～106）个月对29（95％*CI*：6～52）个月，*P*＝0.100］，而在TP53突变亚组中未观察到（图4）。

**图4:**
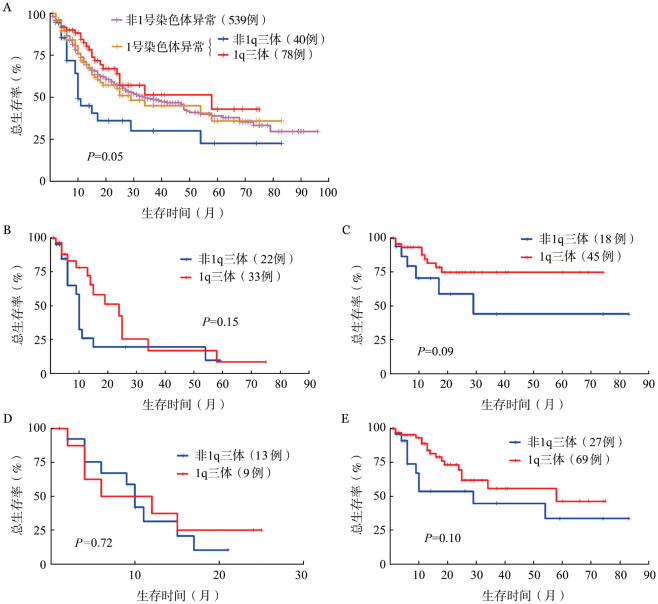
不同亚组骨髓增生异常肿瘤患者总生存曲线比较 **A** 1号染色体异常及非异常患者总生存曲线比较；**B** IB亚组；**C** LB亚组；**D** TP53突变亚组；**E** TP53未突变亚组 **注** LB：骨髓原始细胞<5％或外周血原始细胞<2％；IB：骨髓原始细胞≥5％或外周血原始细胞≥2％或出现Auer小体

三、伴1号染色体异常患者的生存预后影响因素分析

在全部伴1号染色体异常患者中，将年龄、性别、平均红细胞体积（MCV）、IPSS-R预后分层及可能影响生存的基因突变等因素分别行Cox单因素分析，随后将单因素分析中*P*<0.1的因素纳入Cox多因素分析，结果显示IB（*HR*＝2.23，95％*CI*：1.10～4.54，*P*＝0.027）、SF3B1突变（*HR*＝5.61，95％*CI*：2.06～15.29，*P*＝0.001）均是影响伴1号染色体异常患者OS的独立不良预后因素（表2）。

**表2:** 影响伴1号染色体异常骨髓增生异常肿瘤患者总生存的单因素和多因素分析

变量	单因素分析		多因素分析	
	*HR*（95％ *CI*）	*P*值	*HR*（95％ *CI*）	*P*值
年龄≥60岁	2.68（1.46～4.94）	0.002	1.95（0.99～3.83）	0.052
男性	1.01（0.55～1.86）	0.977		
HGB<100 g/L	4.11（1.47～11.50）	0.007	1.35（0.42～4.33）	0.612
ANC<0.8×10^9^/L	1.05（0.57～1.93）	0.882		
PLT≥100×10^9^/L	0.71（0.38～1.32）	0.276		
外周血2～3系减少	3.26（0.64～16.51）	0.154		
1q三体	0.43（0.23～0.78）	0.006	0.68（0.34～1.38）	0.287
IPSS-R中危/高危/极高危	3.35（1.04～10.84）	0.044	1.24（0.34～4.50）	0.743
MCV≤100 fl	3.06（1.58～5.94）	0.001	1.84（0.83～4.07）	0.132
IB	3.44（1.83～6.48）	<0.001	2.23（1.10～4.54）	0.027
骨髓纤维化2～3 级	2.01（1.01～4.01）	0.046	1.77（0.78～4.05）	0.173
复杂核型	2.27（1.24～4.18）	0.008	1.37（0.56～3.40）	0.491
TP53突变	4.32（2.26～8.26）	<0.001	2.55（0.97～6.68）	0.057
RUNX1突变	1.31（0.55～3.11）	0.544		
SF3B1突变	2.36（1.04～5.39）	0.041	5.61（2.06～15.29）	0.001
CBL突变	1.89（0.58～6.13）	0.292		
ASXL1突变	1.21（0.56～2.62）	0.635		
U2AF1突变	0.31（0.08～1.29）	0.107		
DNMT3A突变	3.14（1.08～9.14）	0.036	1.25（0.39～4.03）	0.711
NRAS突变	1.94（0.82～4.60）	0.134		

**注** MCV：平均红细胞体积；IPSS-R：修订的国际预后积分系统；IB：骨髓原始细胞≥5％或外周血原始细胞≥2％或出现Auer小体

## 讨论

本研究我们发现，1q三体在1号染色体异常MDS中最为常见（85例，66.4％），与既往文献报道一致[3],[8]–[9]。本组1q三体患者相较非1q三体具有较低的骨髓原始细胞比例、TP53突变检出率及VAF值。与王炜等[3]报道的1q三体患者中骨髓原始细胞比例较低结果一致。在一项针对1q三体中的一种特殊类型dup（1q）的研究中，相较于其他1号染色体异常患者，dup（1q）组也被观察到具有较低的骨髓原始细胞比例、TP53突变检出率[10]。值的注意的是，MDM4基因位于人类染色体1号染色体的长臂32区（1q32）[11]，MDM4表达会随染色体1q拷贝数增加而增加[12]。Li等[13]从RNA-seq数据中识别出MDM4基因的扩增在肺癌（*OR*＝4.32，*P*<0.001）和乳腺癌（*OR*＝1.46，*P*<0.001）中与TP53突变互斥，与本研究的发现相吻合。未来有必要继续开展在血液肿瘤中的RNA测序研究，以进一步验证这一发现。

Yu等[10]报道了dup（1q）组在1号染色体异常MDS中具有较好的预后，王炜等[3]发现对于LB患者，1q三体组的生存优于非1q三体组，而对于IB患者生存差异无统计学意义。本研究发现相较于非1q三体患者，1q三体组OS时间显著延长。进一步亚组分析显示，在LB、TP53未突变亚组中，1q三体患者OS时间有长于非1q三体患者的趋势，而在IB、TP53突变亚组中未观察到。既往研究表明MDS中TP53突变与急性髓系白血病转化的高风险、对传统疗法的反应不佳和预后不良有关[14]–[16]。本研究的结果提示1q三体患者更好的生存可能与更低的IB、TP53突变检出率且VAF值较低有关，故在IB、TP53突变人群的亚组分析中，1q三体与非1q三体生存无差异。

纳入可能影响生存的多因素校正后显示，1q三体不再为独立的预后保护因素，而IB是独立预后危险因素，与王炜等结果一致[3]。此外发现SF3B1突变是独立不良预后因素。最新版IPSS-M分型明确排除关键高风险事件的SF3B1α亚组具有良好预后，然而携带高风险伴随突变（例如BCOR、BCORL1、NRAS等）的SF3B1β亚组或5q缺失（SF3B15q）的患者预后较差[17]。另有研究通过人源诱导多能干细胞和原代CD34^+^人类造血干细胞模型验证了SF3B1伴随突变会将低风险结局转化为高风险，并发现RUNX1和STAG2的继发突变为主要驱动因素[18]。上述研究部分解释了SF3B1突变作为不良预后因素的原因。目前还没有其他研究指出1号染色体异常核型对SF3B1突变患者的影响，仍有待进一步阐明。

综上，本研究我们发现在伴1号染色体异常MDS患者中，1q三体最为常见且生存较好，其更好的生存可能与其中IB比例、TP53突变率及VAF值更低有关。IB、SF3B1突变是伴1号染色体异常MDS患者预后的独立危险因素。该研究存在以下局限性：①本研究作为一项样本量相对较小的单中心回顾性研究，可能存在选择偏倚，其结论值得在多中心、前瞻性临床试验中进一步验证。②因本研究患者接受的同一药物的药物剂量或疗程、用药依从性差异较大，因此未对该群患者的治疗方案与生存的关联进行分析。③本研究的生存分析对接受移植的患者进行了删失处理，这可能会引入偏倚，独立危险因素在移植后生存中的预后价值受到影响。
